# IGF1 Gene Therapy Modifies Microglia in the Striatum of Senile Rats

**DOI:** 10.3389/fnagi.2019.00048

**Published:** 2019-03-05

**Authors:** Eugenia Falomir-Lockhart, Franco Juan Cruz Dolcetti, Luis Miguel García-Segura, Claudia Beatriz Hereñú, Maria Jose Bellini

**Affiliations:** ^1^Laboratorio de Bioquimica del Envejecimiento, Instituto de Investigaciones Bioquímicas de La Plata (INIBIOLP), Facultad de Ciencias Médicas, UNLP-CONICET, La Plata, Argentina; ^2^Instituto Cajal, CSIC, Madrid, Spain; ^3^Centro de Investigación, Biomédica en Red de Fragilidad y Envejecimiento Saludable (CIBERFES), Instituto de Salud Carlos III, Madrid, Spain; ^4^Instituto de Farmacología Experimental de Córdoba-CONICET, Departamento de Farmacología, Facultad de Ciencias Químicas, UNC-CONICET, Córdoba, Argentina

**Keywords:** IGF1, microglia morphology, striatum, aging, gene therapy

## Abstract

Microglial cells become dystrophic with aging; this phenotypic alteration contributes to basal central nervous system (CNS) neuroinflammation being a risk factor for age related neurodegenerative diseases. In previous studies we have observed that insulin like growth factor 1 (IGF1) gene therapy is a feasible approach to target brain cells, and that is effective to modify inflammatory response *in vitro* and to ameliorate cognitive or motor deficits *in vivo*. Based on these findings, the main aim of the present study is to investigate the effect of IGF1 gene therapy on microglia distribution and morphology in the senile rat. We found that IGF1 therapy leads to a region-specific modification of aged microglia population.

## Introduction

Microglia, the immune cells of the central nervous system (CNS), suffer a phenotypic alteration during aging that is characterized by decreased motility and inefficient surveillance (Streit et al., 2004; von Bernhardi et al., 2016; Koellhoffer et al., 2017). This dystrophic cellular phenotype of microglia is associated with the loss of their neuroprotective function, which contributes to increased basal CNS neuroinflammation with aging and may represent a risk factor for cognitive and motor impairment, depression or diverse age related neurodegenerative diseases (Streit and Xue, 2010; Cunningham, 2013; Patel et al., 2015; Pekny and Pekna, 2016; Ransohoff, 2016; Spittau, 2017).

A feasible approach to modulate microglia function in the aged brain is the use of neurotrophic factors that polarize these cells into a more neurotrophic/neuroprotective phenotype. Among these is insulin like growth factor 1 (IGF1; Arevalo et al., 2010; Suh et al., 2013; Acaz-Fonseca et al., 2014; Labandeira-Garcia et al., 2017), which exerts neuroprotective actions in the CNS (Piriz et al., 2011; Torres Aleman, 2012; Morel et al., 2016), including the aged brain (Piriz et al., 2011; Deak and Sonntag, 2012; Labandeira-Garcia et al., 2017). Previous studies have shown the viability of IGF1 gene therapy to target brain cells *in vivo* (Hereñú et al., 2009) and to decrease behavioral functional impairments in aged rats (Nishida et al., 2011; Pardo et al., 2016). In the present study we report that IGF1 therapy lead to a region-specific modification in microglia number and morphology in the aged brain.

## Materials and Methods

### Adenoviral Vectors

We employed recombinant adenoviral vectors (RAd) previously constructed in our laboratory (Hereñú et al., 2007) as carriers to deliver either the therapeutic cDNA of IGF1 gene (RAd-IGF1) or the red fluorescent protein from Discosoma sp DsRed (RAd-DsRed).

### Animals and Experimental Procedures

Female senile Sprague-Dawley rats (28 months old) were used. These rats have a maximum life expectancy of 36 months (Mansilla et al., 2016). Animals were housed in a temperature-controlled room (22 ± 2°C) on a 12:12 h light/dark cycle and fed *ad libitum*, with a standard chow diet containing 12.08 kJ/g calories: 69.5% from carbohydrates, 5.6% from fat, and 24.9% from protein (Association de Cooperativas Argentinas-S.E.N.A.S.A. No. 04-288/A). All experiments with animals were performed according to the Animal Welfare Guidelines of NIH (INIBIOLP’s Animal Welfare Assurance No A5647-01). The ethical acceptability of the animal protocols used here has been approved by our institutional IACUC (Protocol #T09-01-2013).

On day 0 (D0), rats were anesthetized with ketamine hydrochloride (40 mg/kg; i.p.) plus xylazine (8 mg/kg; i.m.) and placed in a stereotaxic apparatus. Rats were randomly divided into two groups (*n* = 10 per group): DsRed group, which received an injection of RAd-DsRed; and IGF1 group, which received an injection of RAd-IGF1. Bilateral injections in the lateral ventricles were performed placing the tip of a 26 G needle fitted to a 10 μL syringe at the following coordinates relative to the Bregma: −0.8 mm anteroposterior, −4.2 mm dorsoventral and ±1.5 mm mediolateral (Paxinos and Watson, 2007). Rats were injected bilaterally with 8 μL per side of a suspension containing 10^10^ plaque forming units (pfu) of the appropriate vector. Body weight was determined every 2 or 3 days from day −5 before surgery to experimental day 18. Animals were sacrificed at experimental day 18.

### Immunohistochemistry

Animals were placed under deep anesthesia and perfused with phosphate buffered paraformaldehyde 4%, (pH 7.4) fixative. The brains were removed and stored in paraformaldehyde 4%, (pH 7.4) overnight at 4°C. Brains were kept in cryoprotective solution at −20°C until use. For immunohistochemical assessment, brains were cut coronally in 40 μm-thick sections with a Vibratome (Leica).

All immunohistochemical techniques were performed on free-floating sections under moderate shaking. Washes and incubations were done in 0.1 M phosphate buffer pH 7.4, containing 0.3% triton X-100 (washing buffer). The endogenous peroxidase activity was quenched for 15 min at room temperature in a solution of 3% hydrogen peroxide in 50% methanol. After several washes in buffer, sections were incubated overnight at 4°C with an Iba1 rabbit polyclonal antibody diluted 1:1,000 (WAKO CTG2683), marker of microglia/macrophages. Sections were then washed in buffer and incubated for 2 h at room temperature with an anti-rabbit biotinylated secondary antibody (1:1,000, BA-1000; Vector Labs). After several washes in buffer, sections were incubated for 90 min at room temperature with avidin-biotin-peroxidase complex (diluted 1:500, PK-6100; Vector ABC Elite Kit). The reaction product was revealed by incubating the sections with 3,3-diaminobenzidine (Sigma-Aldrich) and 0.01% hydrogen peroxide in 0.1 M phosphate buffer. Then, sections were dehydrated, mounted on gelatinized slides with mounting medium (Vectamount, Vector) and used for image analysis.

### Morphometric Analysis

From each rat, 1 in every 12 brain serial sections was selected. For stereological analysis, we used an Olympus BX-51 microscope attached to an Olympus DP70 CCD video camera (Tokyo, Japan). All morphological parameters were assessed bilaterally. The density of microglia in the striatum (caudate-putamen), dorsal tier of substantia nigra pars compacta (SNCD), motor cortex, dorsolateral entorhinal cortex (DLEnt) and perirhinal cortex (PRh) was assessed. Areas of interest were defined in accordance with the rat brain atlas of Paxinos and Watson (2007). All Iba1-immunoreactive cells were manually quantified, according to the optical disector method, using a counting frame of 83 × 83 μm at 600× magnification. A total of 40–85 counting frames were analyzed. Cells in the uppermost focal plane and/or intersecting the exclusion boundaries of the counting frame were not counted. Cells counts are expressed as number/mm^3^.

Iba1-immunoreactive cells were classified as: (1) non-reactive/ramified microglia; cells with small cell body and few large to no branches; and (2) reactive/ameboid microglia; cells with large or ameboid cell bodies, numerous retracted processes and intense Iba1 immunostaining (Acaz-Fonseca et al., 2015). The proportion of reactive vs. total microglia was determined for each brain region analyzed.

### Statistical Analysis

Data shown in the figures are presented as the mean ± standard error of the mean (SEM). The size of the experimental groups is indicated in each figure legend. Gaussian distribution of data sets was assessed by Kolmogorov-Smirnov test. Statistical analysis was performed by using the software GraphPad Prism 6 (GraphPad Software). To determine significant differences in microglia densities and reactivity between groups, we used *t*-test analysis. *P*-values < 0.05 were considered to be significant.

## Results

### Body Weight Gain

Animal weight was recorded every 2 or 3 days. At day two after administration of RAd-DsRed or RAd-IGF1, both groups experienced a transient loss of body weight due to the surgical procedure. Subsequently, both groups of rats recovered the initial weight and remained stable until the end of the experiment (Supplementary Figure S1).

### Gene Therapy Affects the Number and Reactivity of Microglia

**Figure 1:**
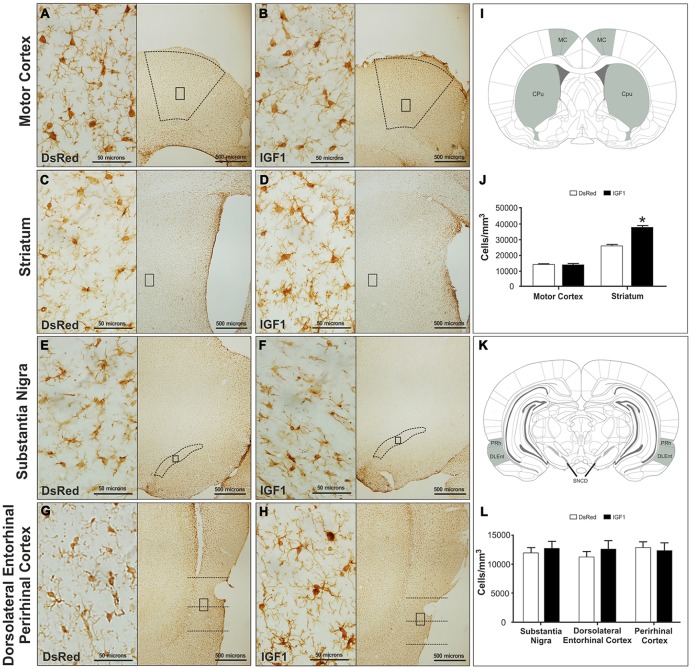
Representative images of motor cortex **(A,B)**, striatum **(C,D)**, substantia nigra compact part dorsal **(E,F)** and dorsolateral entorhinal and perirhinal cortex **(G,H)** of the recombinant adenovirus (RAd)-discosoma Red fluorescent protein (DsRed; **A,C,E,G**) and RAd-Insulin like growth factor 1 (IGF1; **B,D,F,H**) groups at a magnification of 40X (scale bars: 500 microns), with insets at a magnification of 600X (scale bars: 50 microns). Panels (**I**; MC, motor cortex; Cpu, Striatum/caudate-putamen) and (**K**; PRh, perirhinal cortex; DLEnt, dorsolateral entorhinal cortex; and SNCD, substantia nigra compact part dorsal) show a representation of the coronal brain sections where the analyzed regions; are highlighted. Panels **(J,L)** show microglia densities in the different groups in the analyzed regions. Data are given as means ± SEM (*N* = 5/group). *Significant differences (*p* < 0.05).

Iba-1 immunoreactive cells were analyzed in the motor cortex, striatum, substantia nigra, DLEnt and PRh. As shown in Figure 1, significant differences in the number of Iba-1 immunoreactive cells were detected in the striatum between the animals injected with RAd-DsRed and the animals injected with RAd-IGF1. Thus, there was a higher number of Iba-1 immunoreactive cells in the animals injected with RAd-IGF1 than in the animals injected with RAd-DsRed (Figure 1). No significant differences were detected in the number of Iba-1 immunoreactive cells in the motor cortex, substantia nigra and the PRh of the animals injected with RAd-IGF1 and the animals injected with RAd-DsRed (Figure 1).

**Figure 2:**
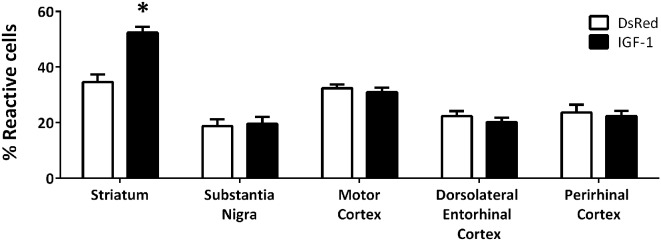
Proportion of reactive microglia with ameboid morphology and enlarged cell body in RAd-DsRed and RAd-IGF1 groups in the different brain regions analyzed. Statistical results indicate significant differences between the experimental groups within the same brain region. Data are given as means ± SEM (*N* = 5/group). *Significant differences (*p* < 0.05).

In addition to the differences in total number, obvious qualitative differences were observed in the morphology of Iba-1 immunoreactive cells between the different experimental groups; with more cells with a reactive phenotype in the striatum of animals injected with Rad-IGF1 (Figure 2). Indeed, the quantitative analysis of reactive/ameboid cells and non-reactive/ramified cells showed a significant increase in the proportion of reactive cells in the striatum of the animals injected with RAd-IGF1 in comparison with the striatum of RAd-DsRed injected rats. In contrast, no significant differences in the proportion of reactive and non-reactive phenotypes were detected in the other brain regions analyzed (Figure 2).

In summary, these findings indicate that the injection of RAd-IGF1 is able to increase the number of Iba-1 immunoreactive cells and the proportion of Iba-1 immunoreactive cells with a reactive phenotype in a specific brain region.

## Discussion

We previously demonstrated that ICV IGF1 gene therapy is effective to transduce brain ependymal cells with high efficiency, achieving effective release of transgenic IGF1 into the cerebrospinal fluid (CSF; Hereñú et al., 2009) and restoring motor performance in aged animals (Nishida et al., 2011). Thus, we decided to use the ependymal route to implement IGF1 gene therapy in aging rats with the goal to characterize microglia number and distribution in different brain regions related with cognition or motor performance.

ICV gene therapy caused a significant but transient decrease in weight gain in both DsRed and IGF1 groups. Body weigh may be affected by surgery and also by a temporary induction of endogenous neurotrophic factors, such as GDNF, which has been reported to cause body weight loss (Morel et al., 2010). This effect could be greater in the IGF1 group, since IGF1 may regulate the hypothalamic system controlling body weight and energy expenditure (Werner and LeRoith, 2014).

We expected that IGF1 gene therapy will decrease microglia reactivity, since this therapy has been shown to reduce the reactivity of astrocytes in response to proinflammatory stimuli *in vitro* (Bellini et al., 2011). And to exert neuroprotective and neuroreparative actions in experimental animal models of stroke (Zhu et al., 2008; Liu et al., 2017). However, other studies have reported detrimental actions of IGF1 receptor signaling in the brain of mouse models of Alzheimer’s disease (Cohen et al., 2009; Freude et al., 2009; Gontier et al., 2015). In this study we found is that IGF1 gene therapy increased the number and the proportion of microglia with a reactive phenotype in a region dependent manner.

We have not a definitive explanation for the regional effect of RAd-IGF1 on striatal microglia. It is well established that microglia (Breese et al., 1996; Walter et al., 1999; Chesik et al., 2004; Suh et al., 2013; Rodriguez-Perez et al., 2016; Trueba-Saiz et al., 2017) and other cell types in the brain (Fernandez and Torres-Alemán, 2012) express IGF1 receptors. It is possible that regional differences in the expression of these receptors may cause a different sensitivity to IGF1 released and delivered to the ventricles by infected ependymal cells.

The functional consequences of these changes in microglia are unknown. However, it has been described that treatments that leads to the depletion of microglia in injury models increases neuronal death (Vinet et al., 2012). Reciprocally, repletion of microglia or introduction of exogenous microglia result in neuronal rescue, driven possibly by microglial production of trophic growth factors, or *via* the clearance of deleterious byproducts of metabolism and neurotransmission (Streit et al., 2004; Nissen, 2017). Therefore, it is possible that the increased number of microglia cells induced by IGF1 gene therapy may exert a protective function in the striatum of older rats. Indeed, previous studies have shown that the administration of RAd-IGF1 in the brain, following the protocol used in the present study, results in an improvement in motor function of senile rats (Nishida et al., 2011).

In summary, our findings indicate that ICV IGF1 gene therapy specifically modifies the number and phenotype of microglia in the striatum of senile rats, suggesting that such a therapy may be useful to alter the function of dystrophic microglia, at least in specific brain regions. Further studies should determine whether the effect of IGF1 gene therapy on microglia is age-dependent and is also detected in male animals.

## Data Availability

All datasets generated for this study are included in the manuscript and/or the supplementary files.

## Author Contributions

EF-L and MB designed the experiments. EF-L and FD performed the experiments. EF-L, FD, LG-S, CH and MB analyzed the data. EF-L, LG-S, CH and MB wrote the manuscript.

## Conflict of Interest Statement

The authors declare that the research was conducted in the absence of any commercial or financial relationships that could be construed as a potential conflict of interest.

## Supplementary Material

FIGURE S1 Time curve of body weight gains of RAd-DsRed and RAd-IGF1 rats. *Significant difference (*p* < 0.05). *N* = 5/group.

## Abbreviations

IGF1: insulin like growth factor 1
RAd: recombinant adenovirus
DsRed: Discosoma Red fluorescent protein
CNS: central nervous system.
