# Supplementary figures and images for: IGF1 Gene Therapy Modifies Microglia in the Striatum of Senile Rats
Source: Front Aging Neurosci. 2019 Mar 5;11:48. doi: 10.3389/fnagi.2019.00048 (PMC6411822; doi:10.3389/fnagi.2019.00048)

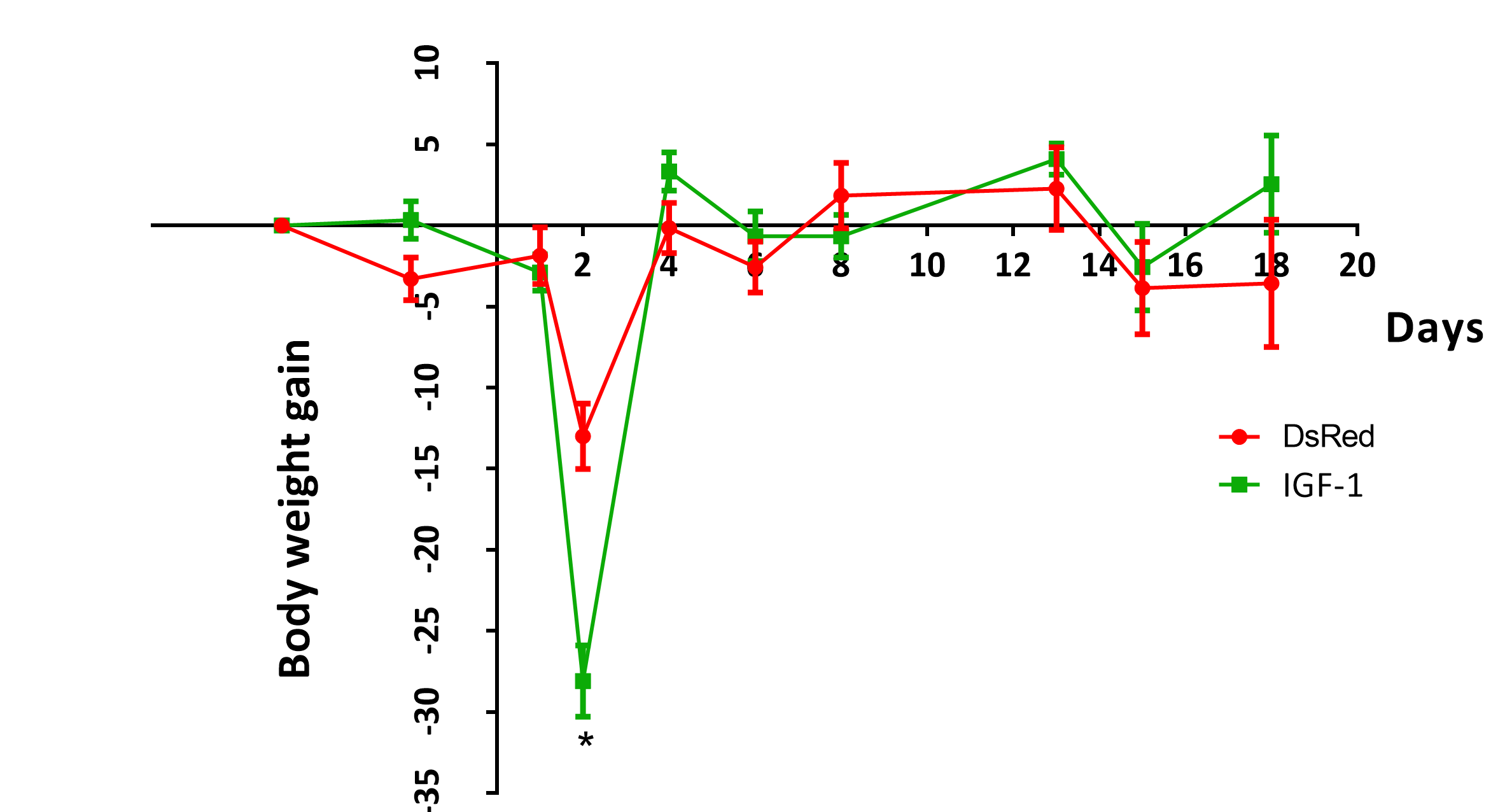

Supplement: FIGURE S1 — Time curve of body weight gains of RAd-DsRed and RAd-IGF1 rats. *Significant difference (p < 0.05). N = 5/group. [file Image_1.tif]
